# Individual Differences in Ethanol Drinking and Seeking Behaviors in Rats Exposed to Chronic Intermittent Ethanol Vapor Exposure is Associated with Altered CaMKII Autophosphorylation in the Nucleus Accumbens Shell

**DOI:** 10.3390/brainsci9120367

**Published:** 2019-12-11

**Authors:** Sucharita S. Somkuwar, Chitra D. Mandyam

**Affiliations:** 1 VA San Diego Healthcare System, San Diego, CA 92161, USA; sucharita.somkuwar@gmail.com; 2 Department of Anesthesiology, University of California San Diego, San Diego, CA 92161, USA

**Keywords:** high responders, low responders, nucleus accumbens shell, CaMKII, CIE, ethanol self-administration

## Abstract

Chronic intermittent ethanol vapor exposure (CIE) in rodents produces reliable and high blood ethanol concentration and behavioral symptoms associated with moderate to severe alcohol use disorder (AUD)—for example, escalation of operant ethanol self-administration, a feature suggestive of transition from recreational to addictive use, is a widely replicated behavior in rats that experience CIE. Herein, we present evidence from a subset of rats that do not demonstrate escalation of ethanol self-administration following seven weeks of CIE. These low responders (LR) maintain low ethanol self-administration during CIE, demonstrate lower relapse to drinking during abstinence and reduced reinstatement of ethanol seeking triggered by ethanol cues when compared with high responders (HR). We examined the blood ethanol levels in LR and HR rats during CIE and show higher levels in LR compared with HR. We also examined peak corticosterone levels during CIE and show that LR rats have higher levels compared with HR rats. Lastly, we evaluated the levels of Ca^2+^/calmodulin-dependent protein kinase II (CaMKII) in the nucleus accumbens shell and reveal that the activity of CaMKII, which is autophosphorylated at site Tyr-286, is significantly reduced in HR rats compared with LR rats. These findings demonstrate that dysregulation of the hypothalamic–pituitary–adrenal axis activity and plasticity-related proteins regulating molecular memory in the nucleus accumbens shell are associated with higher ethanol-drinking and -seeking in HR rats. Future mechanistic studies should evaluate CaMKII autophosphorylation-dependent remodeling of glutamatergic synapses in the ventral striatum as a plausible mechanism for the CIE-induced enhanced ethanol drinking and seeking behaviors.

## 1. Introduction

Progressive escalation of voluntary alcohol intake is a hallmark of moderate to severe alcohol use disorder (AUD). The Diagnostic and Statistical Manual-V (DSM V) presents AUD criteria like drinking “more or longer that you intended” and could not “cut down or stop drinking” which may be interpreted as a description of escalation [1]. It is not surprising, therefore, that escalation of voluntary alcohol intake is considered a major face validity criteria for any animal model of AUD [2]. As a consequence, such models can provide valuable information about why only a relatively small percentage of people drinking alcohol develop AUD [3,4,5]. Based on several clinical studies, it can be confirmed that prolonged alcohol use, genetic factors, psychosocial, cognitive, and environmental risk factors could play a role in the individual variability in the development of moderate to severe AUD [4,6,7,8]. Understanding the mechanisms that underlie individual vulnerability to moderate to severe AUD may help enhance the number of effective treatment strategies for AUD and reduce the economic burden associated with the disorder [9,10].

Several rodent lines have been bred for their differences in ethanol consumption [11,12,13,14]. Furthermore, individual differences in operant ethanol self-administration have been documented in outbred rodents [15,16,17], and have been related to individual differences in ethanol reinforcement, motivation to obtain ethanol and AUD-like behavior. More notable is that, in these studies, AUD-like behavior in selected high- versus low-ethanol drinking rats (high responders, HR; low responders, LR) facilitated the investigation of the neurobehavioral mechanisms underlying the individual risk for AUD [16]. 

Ca^2+^/calmodulin-dependent protein kinase II (CaMKII) is strongly implicated in the induction and maintenance of synaptic strengthening via autonomous activity and increases in autophosphorylation at T286 [18,19,20]. Mechanistic and correlative studies have demonstrated a role of CaMKII autophosphorylation in alcohol addiction-related behaviors [21,22,23,24,25]. For example, even though CaMKII autophosphorylation-deficient mice self-administer similar amounts of ethanol in an operant paradigm, they do not show psychomotor responses to acute and chronic alcohol injections during an ethanol-free state or during voluntary ethanol-drinking states [23,24]. Furthermore, CaMKII autophosphorylation-deficient mice demonstrate enhanced negative reinforcing actions of ethanol [25], suggesting a role for CaMKII autophosphorylation in the negative affective state observed in chronic intermittent ethanol vapor exposure (CIE) rats. In support of this hypothesis, recent studies indicate that neural activity in the ventral striatum is critical for the neurochemical effects of ethanol, which may contribute to further excessive ethanol consumption and dependence [26,27]. More notable is that reduced activity of CaMKII at its autophosphorylation site was observed in the ventral striatum, selectively in the nucleus accumbens shell region, following chronic ethanol drinking [21]. The glutamate (N-methyl-d-aspartate, NMDA) receptors (GluNs) are the targets for the inhibitory actions of ethanol and play a role in alcohol dependence [28,29,30,31]. In particular, the modulation of GluN2A activity may be responsible for the hyper-excitability of the brain during ethanol withdrawal and may represent one mechanism involved in ethanol relapse behavior [32]. Phosphorylation of GluN2A, particularly at Tyr-1325, is known to enhance GluN2A activity [33], and the activation of GluN2A further enhances CaMKII autophosphorylation [34]. To date, however, relatively little information is available concerning the activity of CaMKII and GluN2A in the ventral striatum and its association with individual differences in operant ethanol self-administration in outbred rodents in a model of moderate to severe AUD. Our study, therefore, proposed to observe and document these changes, following CIE in HR and LR rats.

## 2. Materials and Methods

### 2.1. Animals

Fifty-one adult male Wistar rats (Charles River Laboratories, Wilmington, MA, USA) completed the study. All rats were eight weeks old at the beginning of the study, and weighed approximately 220–250 g. The rats were maintained in reverse 12 h light–12 h dark cycle rooms and housed two/cage unless otherwise specified. Food and water were available ad libitum. All experimental procedures were carried out in strict adherence to the National Institutes of Health Guide for the Care and Use of Laboratory Animals (NIH publication number 85–23, revised 1996), and were approved by the Institutional Animal Care and Use Committee at Scripps Research.

### 2.2. Ethanol Self-Administration

Forty-four adult male Wistar experimentally-naïve rats completed the study at the same time in two separate cohorts. Rats were separated into high responders (HR) and low responders (LR) based on their drinking data. Operant behavior data, blood ethanol levels (BELs; [35]) and peak corticosterone levels [36] of twenty-one HR rats have been reported elsewhere. Behavior data, BELs and corticosterone data for twenty-three LR rats have never been published previously, and are compared with the previously published findings and reported here. Operant behavior data, BELs, corticosterone data and Western blotting data for HR and LR rats were analyzed at the same time. Rats were given two 14 h lever-responding training sessions in the operant conditioning boxes (Med Associates Inc., Fairfax, VT, USA), on a fixed-ratio one schedule (FR1; one response resulted in one reinforced delivery), where one press on the available lever resulted in the delivery of 0.1 mL of water to a sipper cup mounted on the wall in between the two levers. The operant conditioning boxes were housed inside sound-attenuating chambers. During these sessions, the house-light and white noise were turned off (Context A). Then, rats were trained to respond for 0.1 mL of ethanol (10% v/v) over four daily 2 h FR1 sessions; all other conditions remained the same as before. Subsequently, the rats were trained to discriminate between two available levers to obtain 0.1 mL ethanol during daily 30 min FR1 sessions. During these sessions, active (right) lever responding resulted in the delivery of ethanol, while responding on the inactive (left) lever was recorded but had no programmed consequence. Each ethanol delivery was followed by a 4 s time-out, during which responding on the active lever did not result in the delivery of ethanol. During this time-out period, the cue-light above the active lever remained on; thus, the cue-light was paired with the delivery of ethanol. These 30 min discrimination training sessions continued till stable responding was obtained, where stable responding was defined as less than 10% variation in active lever responding for three consecutive 30 min FR1 sessions. Subsequently, all the rats experienced chronic intermittent ethanol vapor exposure (CIE; described below) for a duration of seven weeks. All rats continued to experience two 30 min FR1 sessions per week (Tuesdays and Thursdays) during the seven weeks of vapor exposure (maintenance). Responding was analyzed to determine escalation of self-administration compared to pre-vapor stable responding. After seven weeks of CIE, rats were withdrawn from CIE. 

### 2.3. Chronic Intermittent Ethanol Vapor Exposure (CIE)

During CIE, rat cages were housed in specialized chambers and were exposed to alcohol vapors on a 14 h ON/10 h OFF schedule. Alcohol (95% ethanol) from a large reservoir was delivered to a heated flask at a regulated flow rate using a peristaltic pump (model QG-6, FMI Laboratory, Fluid Metering Inc., Syosset, NY, USA). The drops of alcohol in the flask were immediately vaporized and carried to the vapor chambers containing the rat cages by controlled air flow (regulated by a pressure gauge). The air pressure and ethanol flow rates were optimized to obtain BELs between 125 and 250 mg/dL or 27.2 and 54.4 mM.

### 2.4. Tail Bleeding for Determination of BEL

For measuring BELs, tail bleeding was performed in all rats, once a week (every Friday), between 13 and 14 h of vapor exposure. Rats were gently restrained while the tip of the tail was pricked with a clean needle. Tail blood (0.2 mL) was collected and centrifuged at 2000 rpm for 10 min. Plasma (5 uL) was used for measurement of BELs using an Analox AM1 analyzer (Analox Instruments, Lunenburg, MA, USA). Single-point calibrations were performed with alcohol (100 mg/dL) for each set of samples with reagents provided by Analox Instruments. When plasma alcohol levels were outside the target range (125–250 mg/dL), ethanol vapor flow was adjusted accordingly.

### 2.5. Drinking During Abstinence (DDA)

After 23 days of abstinence from CIE and ethanol self-administration, all rats were given one 30 min FR1 session to lever press for ethanol reinforcement (0.1 mL of ethanol) under cue–context conditions identical to those used for training and maintenance. Active and inactive lever responses were recorded.

### 2.6. Extinction

Following DDA, rats were subject to six daily 30 min extinction sessions under a different cue–context combination than that used for training and maintenance (Context B). Specifically, operant boxes different from those used for self-administration were used and the house-light and white noise were turned on, and no cue lights were available following lever presses. Finally, lever response did not result in the delivery of ethanol. Both lever responses were recorded.

### 2.7. Cue-Induced Reinstatement

Following the sixth day of extinction, rats were subject to one session of cued-context reinstatement of ethanol seeking. Specifically, rats were introduced to operant chambers under conditions identical to training and maintenance (no house-light, no white noise; Context A). Active lever responses resulted in the presentation of the cue-light for 4 s, but did not result in the delivery of ethanol. Both active and inactive lever responses were recorded.

### 2.8. Plasma Corticosterone Quantification

Plasma corticosterone was measured using the DetectX ^®^ Corticosterone Enzyme Immunoassay Kit (Arbor Assays, Ann Arbor, MI, USA) following manufacturer instructions. Plasma collected for measuring BELs was used to determine peak corticosterone levels. On the day of the assay, the plasma of a subset of HR and LR rats was allowed to thaw on ice and the reagents of the Enzyme Immunoassay Kit were allowed to warm to room temperature prior to use. Samples were prepared by mixing plasma with the Dissociation Reagent in a 1:1 ratio, and then diluting the mixture with Assay Buffer to get a final dilution of 1:100 for the plasma. The manufacturer-provided corticosterone standard (100 ng/mL) was serially diluted to generate an 8-point standard curve ranging from 78.125 to 10,000 pg/mL. Standards and the diluted samples were added to a microtiter plate coated with a secondary antibody against sheep. A sheep polyclonal antibody against corticosterone and corticosterone-peroxidase conjugate were added to sample and standard wells. Following an hour of incubation, binding of the corticosterone and corticosterone-peroxidase conjugate to the plate was stopped by washing the wells. Then, a peroxidase substrate was added to the wells that produced a colorimetric reaction with the bound corticosterone–peroxidase conjugate. This reaction was stopped, and the intensity of the generated color (or optical density) was measured at 450 nm using a microtiter plate reader (Biotek Synergy, Winooski, VT, USA). The concentration of corticosterone in the samples was calculated from the 4-parameter logistic non-linear regression obtained from the concentration–optical density plot generated by the eight known standard dilutions using Prism 7 (GraphPad Software, Inc., San Diego, CA, USA). 

### 2.9. Brain Tissue Collection and Western Blotting

Fourteen CIE rats (*n* = 8 HR and *n* = 6 LR) were euthanized between 45 min and 1 h after the reinstatement session, and seven CIE-naïve control rats, age-matched, were euthanized at the same time by rapid decapitation, and the brains were isolated, and dissected along the midsagittal plane. The left hemisphere was snap frozen for Western blotting analysis. Western blot procedures optimized for measuring levels of both phosphoproteins and total proteins were employed [37,38]. Tissue punches (0.75 mm internal diameter, model # PUN0750, Zivic Instruments, Pittsburgh, PA, USA) from 2 300 um thick sections of the ventral striatum (2.7 to 1.7 mm from bregma), mostly containing the nucleus accumbens shell, were homogenized by sonication in ice-cold buffer (320 mM sucrose, 5 mM HEPES, 1 mM EGTA, 1 mM EDTA, 1% SDS, with Protease Inhibitor Cocktail and Phosphatase Inhibitor Cocktails II and III diluted 1:100; Sigma, St. Louis, MO, USA), and protein concentration was determined using a detergent-compatible Lowry method (Bio-Rad, Hercules, CA, USA). A total of 20 ug protein samples were subjected to gel electrophoresis and transferred to PVDF membranes. The membranes were incubated with total (t)CaMKII (rabbit polyclonal, 1:200, Abcam cat# ab52476, molecular weight 47 and 55 kDa), phosphorylated (p)CaMKII Tyr-286 alpha (rabbit polyclonal, 1:200, Abcam cat# ab5683, molecular weight 50 kDa); antibody to glutamate (NMDA) receptor subunit 2A (tGluN2A; rabbit polyclonal, 1:200, Santa Cruz Biotechnology cat# sc-9056, molecular weight 170 kDa), pGluN2A Tyr-1325 (rabbit polyclonal, 1:200, PhosphoSolutions, cat# P1514–1325, molecular weight 180 kDa). Blots were then washed three times for 5 min in 1x tris-buffered saline, 0.1% tween-20 (TBST), and then incubated for 1 h at room temperature with horseradish–peroxide conjugated goat antibody to rabbit in TBST. Following subsequent washes, immunoreactivity was detected using SuperSignalWest Dura chemiluminescence detection reagent (Thermo Scientific, Waltham, MA, USA) and images were collected using a digital imaging system (Azure Imager c600, VWR, Radnor, PA, USA). For normalization purposes, membranes were incubated with 0.125% coomassie stain for 5 min and washed three times for 5–10 min in de-stain solution [39,40]. Densitometry was performed using ImageJ software (NIH). The signal value of the band of interest following subtraction of the background calculation was then expressed as a ratio of the corresponding coomassie signal (following background subtraction). This ratio of expression for total protein (alpha for CaMKII) was then expressed as a percent of the control sample included on the same blot. For analysis of phosphorylated proteins, the ratio of expression of phosphorylated protein to the total protein was first calculated and then expressed as a percent of the control sample included on the same blot.

### 2.10. Statistical Analysis

Ethanol intake prior to and during CIE was compared using repeated measures 2-way ANOVA, with time in weeks as the within-subjects factor and groups (LR, HR) as the between-subjects factor. Further, active lever responding during extinction and reinstatement were compared as repeated measures 2-way ANOVA, with session (extinction and reinstatement) as the within-subjects factor and groups (HR, LR) as the between subjects factor. Separate paired t-tests were conducted for HR and LR rats to compare differences in responding on the active and inactive levers during reinstatement. BELs and peak corticosterone were evaluated using repeated measures 2-way ANOVA, with time (seven levels during CIE and three levels during abstinence) as the within-subjects factor and treatment (LR, HR) as the between-subjects factor. Protein expressions, quantified as % age-matched ethanol and behaviorally naïve controls, were compared using one-way ANOVA, with groups as between-subjects factors. Tukey’s post-hoc analyses were used to further probe significant main effects and interactions in ANOVAs. Significance was determined at *p* < 0.05. All statistical analyses were conducted using Prism 7 (GraphPad Software, Inc., San Diego, CA, USA).

## 3. Results

### 3.1. Escalation of Ethanol Drinking 

**Figure 1:**
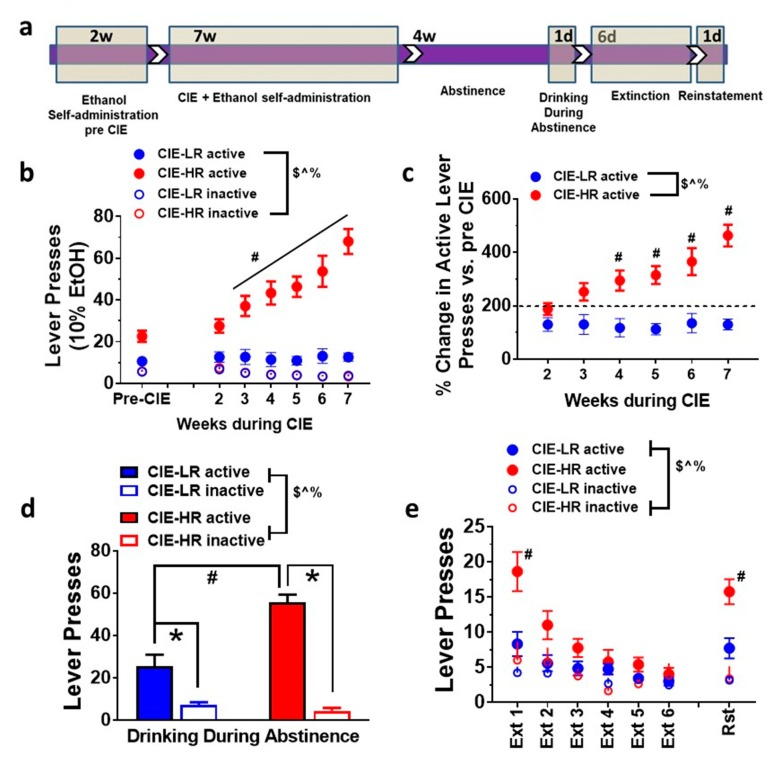
Individual differences in operant responses in ethanol drinking and seeking behaviors in low responder (LR) and high responder (HR) rats. (**a**) Schematic of the experimental design indicting the order of behavioral studies and time frame in each sub paradigm. w, weeks; d, days. (**b**) Active and inactive lever responses in LR and HR rats during pre-vapor sessions and during chronic intermittent ethanol vapor exposure (CIE) sessions. (**c**) Data are extrapolated from panel (**b**) and represented as percent change in active lever responses from pre-vapor session. (**d**) Active and inactive responses in LR and HR rats from drinking session conducted during abstinence. (**e**) Active and inactive lever responses in LR and HR rats from extinction and contexual cued reinstatement session. Data are expressed as mean ± SEM. ^$^ *p* < 0.05 group × weeks interaction, ^^^ *p* < 0.05 main effect of weeks of CIE, ^%^ *p* < 0.05 main effect of group. ^#^ *p* < 0.05 vs. LR, * *p* < 0.05 vs. inactive lever responses within groups by post hoc analysis. *n* = 23 LR, *n* = 21 HR in figures b-d; *n* = 23 LR, *n* = 8 HR in figures d-e.

Rats were separated into HR and LR based on escalation criteria (defined as >150% change in active lever (reinforced) responses during CIE compared with pre-CIE sessions, after a median split analysis on their reinforced lever responses during CIE sessions; Figure 1c). Repeated measures two-way ANOVA indicated that HR have higher responding on reinforced (active) levers, indicated by a significant group × weeks interaction F (6,252) = 16.55, main effect of the group F (1,42) = 45.24 and main effect of weeks F (6,252) = 18.42, *p* < 0.0001 (Figure 1b). Repeated measures two-way ANOVA did not detect an interaction F (6,252) = 0.2218, or main effect of group F (1,42) = 0.02436; however, it detected a main effect of weeks F (6,252) = 14.12, *p* < 0.0001, when the non-reinforced (inactive) levers were analyzed (Figure 1b). HR demonstrated a higher percent change in active lever responses during weeks of CIE compared with pre-CIE sessions, when compared with LR rats (significant interaction F (5,210) = 11.04, effect of group F (1,42) = 22.31 and weeks F (5,210) = 11.45, *p* < 0.0001; Figure 1c) by repeated measures two-way ANOVA.

### 3.2. Blood Ethanol Levels during CIE

**Figure 2:**
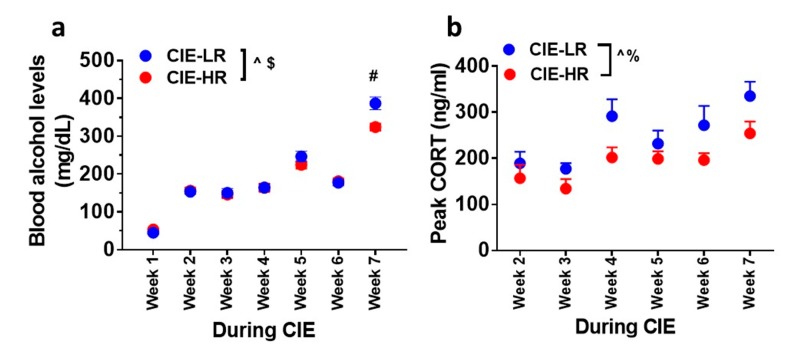
LR and HR rats differ in plasma BELs and peak corticosterone levels during CIE. (**a**) Plasma BELs expressed as mg/dL during weeks of CIE. (**b**) Plasma peak corticosterone levels expressed as ng/mL during weeks of CIE. Data are expressed as mean ± SEM. *^$^ p* < 0.05 group x weeks interaction, ^^^ *p* < 0.05 main effect of weeks of CIE, ^%^ *p* < 0.05 main effect of group. ^#^ *p* < 0.05 vs. HR by post hoc analysis. *n* = 12 LR, *n* = 20 HR for plasma BELs. *n* = 6 LR, *n* = 9 HR for peak corticosterone levels.

The amount of ethanol experienced by all rats during weeks of CIE reached ≥150 mg/dL of plasma (Figure 2a). Repeated measures two-way ANOVA detected a significant interaction F (6,180) = 4.1, *p* = 0.007, and a main effect of weeks F (6,180) = 235.6, *p* <0.001, and did not detect a main effect of groups F (1,30) = 3.2, *p* = 0.07. Posthoc analysis showed higher levels of BELs in LR rats compared with HR rats during Week 7 (*p* < 0.05; Figure 2a). 

### 3.3. Plasma Corticosterone Levels during CIE

Peak plasma corticosterone levels were measured during weeks of CIE. Repeated measures two-way ANOVA did not detect a significant interaction F (5,65) = 0.5869, *p* = 0.7; however, it detected a main effect of weeks F (5,65) = 9.069, *p* <0.001, and a main effect of groups F (1,13) = 8.437, *p* = 0.01; Figure 2b.

### 3.4. Drinking during Abstinence

Following four weeks of abstinence, HR demonstrated higher drinking (higher active lever responses) compared to LR when rats were given access to ethanol. Two-way ANOVA detected a significant group x lever interaction F (1,24) = 24.7, *p* < 0.001; main effect of group F (1,24) = 17.47, *p* = 0.003; and main effect of lever F (1,24) = 109.9, *p* < 0.001; Figure 1d. Posthoc analysis showed higher active lever responses in LR and HR rats versus inactive lever responses, and higher active lever responses in HR rats versus LR rats (*p* < 0.05).

### 3.5. Extinction and Contextual Cued Reinstatement of Ethanol Seeking

Following drinking during abstinence, the rats were subjected to six sessions of extinction in a novel context (Context B, different from self-administration Context A). HR rats demonstrated enhanced seeking during extinction compared to LR rats. Repeated measures two-way ANOVA indicated a significant group x days interaction F (5,125) = 7.6, *p* < 0.001; main effect of groups F (1,5) = 5.6, *p* < 0.02; and main effect of days F (5,125) = 30.9, *p* < 0.001; Figure 1e. Posthoc analysis showed higher lever responses on the previously paired active lever on the first session of extinction in HR rats versus LR rats (*p* < 0.05). All rats extinguished prior to reinstatement testing.

Following extinction sessions, the rats were subjected to contextual cued reinstatement (in Context A, where active lever responding with cues is indicative of delivery of alcohol). Reinstatement of ethanol-seeking on the previously paired active lever was higher in HR rats compared to LR rats. Repeated measures two-way ANOVA indicated a significant group × days interaction F (1,25) = 11.41, *p* = 0.02; main effect of groups F (1,25) = 7.754, *p* < 0.01; and main effect of days F (1,25) = 62.4, *p* < 0.001; Figure 1e. Posthoc analysis showed higher lever responses on the previously paired active lever during cue-induced reinstatement in HR rats versus LR rats (*p* < 0.05, Figure 1e).

### 3.6. Expression of Plasticity-Related Proteins in the Nucleus Accumbens Shell of the Ventral Striatum 

**Figure 3:**
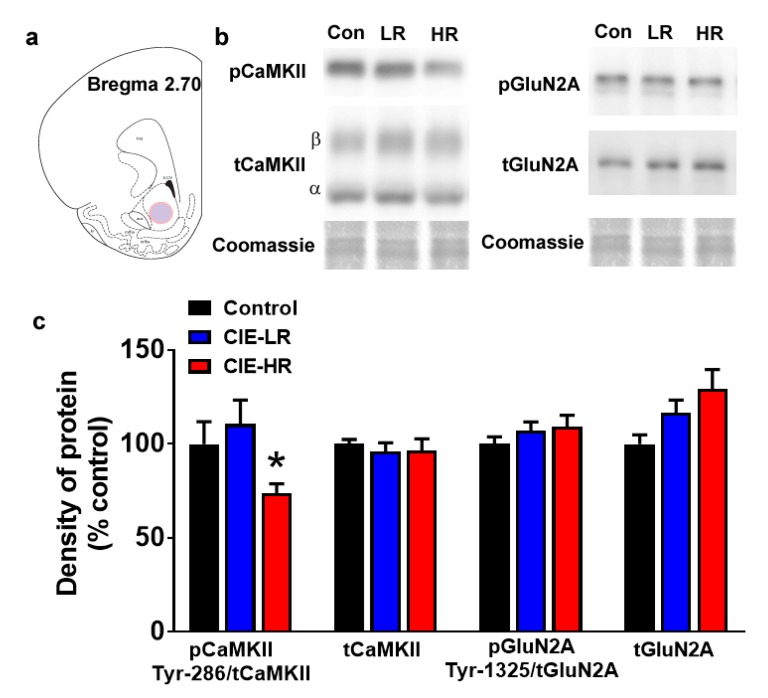
LR and HR rats differ in the density of pCaMKII in the nucleus accumbens shell region. (**a**) Schematic of a coronal section through the rat brain, showing the location of tissue punch (colored area) taken in the nucleus accumbens shell. (**b**) Representative immunoblots of proteins from one control (con), LR and HR rat. Coomassie staining is indicated as a loading control for each sample. (**c**) Quantitative analysis of proteins from control, LR and HR rats. *n* = seven controls, *n* = 6 LR and *n* = 8 HR. Data are expressed as mean ± SEM. * *p* < 0.05 vs. LR by One-Way ANOVA followed by post hoc analysis.

Western blotting was performed to determine quantitative differences in activated and total CaMKII, and activated and total GluN2A. One-way ANOVA of pCaMKII demonstrated reduced expression (F (2,18) = 3.74, *p* = 0.04; Figure 3a,b), and posthoc analysis revealed reduced pCaMKII expression in HR rats compared to LR rats (*p* < 0.05). No significant changes were observed in tCaMKII, tGluN2A and pGluN2A. 

## 4. Discussion

The first goal of the study was to identify individual differences in ethanol-drinking behaviors during CIE and ethanol-seeking behaviors during abstinence from chronic ethanol experience in outbred Wistar rats. The second goal was to determine the associated changes in plasticity-related proteins, especially CaMKII, given the role of this protein in regulating alcohol drinking behaviors. Here, we report that outbred Wistar rats demonstrate marked individual differences in ethanol self-administration during CIE. A subgroup of the CIE rats, HR rats, escalated their ethanol intake. Moreover, HR showed a higher ethanol consumption during protracted abstinence, and enhanced ethanol-seeking during extinction and cue-induced reinstatement. These findings show that HR develop characteristics of addiction-like behavior, a hallmark of moderate to severe AUD in humans. Neurobiological changes that coincided with enhanced ethanol-seeking triggered by ethanol context and cues were examined in the nucleus accembens shell, and we report that the activity of CaMKII at its autophosphorylation site was significantly reduced in HR rats compared with LR rats. More importantly, the effects observed with CaMKII were not generalized to other plasticity-related proteins, including GluN2A, indicating that reduced phosphorylation is specific to CaMKII. These results indicate that the CaMKII autophosphorylation may play an important role in regulating highly motivated alcohol seeking in HR rats, even when alcohol is not available. 

CIE has been successfully used to produce alcohol dependence in rodents, measured by the ability of this procedure to produce somatic withdrawal symptoms and negative affective behavior in the absence of alcohol, and motivation to seek ethanol in otherwise healthy animals [17]. More notable is that CIE produces blood- and brain-ethanol levels that positively correlate, and provides evidence for the development of metabolic tolerance to alcohol vapor with repeated exposure [17,41]. Furthermore, metabolic tolerance has been linked to functional tolerance, seen as enhanced operant self-administration or escalation of ethanol self-administration in CIE rats [17]. We separated outbred rats into HR and LR based on their escalation of ethanol drinking after approximately three weeks of CIE. Interestingly, although these two groups showed a nonsignificant trend in differences in ethanol drinking before the initiation of CIE, they began to diverge dramatically two weeks into CIE, and this pattern persisted for the remaining weeks of CIE. We measured BELs during CIE in both groups, and demonstrate that this method of induction produced higher levels of BELs in the LR, at least during the last week of CIE. The differences in BELs may reflect differences in alcohol pharmacokinetics [42], including elimination rate [43]. In addition, differences in BELs may also reflect differences in basal body temperature, as lower temperatures correlate with a lower elimination rate [44,45,46]. While these issues were not investigated in the current study, they are important future pursuits. Nevertheless, these results indicate that one of the main determinants of individual differences in ethanol self-administration in outbred rats is the amount of effort or motivation required to acquire ethanol, and could emphasize that individual differences in withdrawal-induced ethanol drinking in CIE rats could be based on their propensity to exert effort for ethanol reward and not by the BELs maintained during CIE. 

A compromised hypothalamic–pituitary–adrenal (HPA) axis function has become a valid feature underlying moderate to severe AUD [36,47,48,49,50]. For example, baseline and peak corticosterone were higher in rats that did not demonstrate escalation of ethanol self-administration compared with rats that showed escalation in ethanol self-administration after ethanol challenge [47]. These findings reveal that escalation of ethanol drinking resulted in significant impairment of HPA function. Our results of higher peak corticosterone in LR rats versus HR rats extend these findings and show that peak corticosterone levels are blunted in HR rats that consume higher amounts of ethanol under the influence of CIE. 

With respect to the reinforcing properties of ethanol, behavioral studies demonstrate that Wistar rats directly self-administer ethanol in vivo into the shell region of the nucleus accumbens [51]. More notable is that the reinforcing effects of ethanol were not generalized to other regions in the ventral striatum, as Wistar rats did not self-administer ethanol into the core region of nucleus accumbens [51]. Furthermore, the increased sensitivity of the nucleus accumbens shell to the reinforcing properties of ethanol is observed in alcohol-preferring P rats that are selectively bred for high alcohol intake [51]. Mechanistic studies have also supported the role of nucleus accumbens shell in the context-driven reinstatement of ethanol-seeking [52,53]. These findings suggest that alterations in the expression of plasticity-related proteins in the nucleus accumbens shell could be predicted by the enhanced reinstatement of ethanol-seeking in HR rats. In support of this, a recent study demonstrated that chronic ethanol consumption produces profound alterations in proteins associated with neuronal signaling in the nucleus accumbens shell, with lesser effects in the nucleus accumbens core region [21]. Particularly interesting was the reduced activity of CaMKII at its autophosphorylation site after a chronic ethanol liquid diet in the nucleus accumbens shell region. Our results extend these findings and demonstrate that the activity of CaMKII at its autophosphorylation site is reduced in HR rats compared with LR rats in the nucleus accumbens shell region. Given that CaMKII autophosphorylation contributes to a variety of neuroadaptations in the brain, and particularly in the striatum, including the remodeling of glutamatergic neurotransmission, activity-dependent trafficking of PSD-95, expression of neuronal-specific nitric oxide synthase and the generation of free radicals, and plays a significant role in learning and memory functions dependent on the striatum [54,55,56,57,58,59,60], we speculate that the possible mechanism of enhanced ethanol-seeking in HR rats involves altered glutamatergic signaling, oxidative stress and altered learning and memory functions induced by ethanol via reduced CaMKII autophosphorylation. Further studies are, however, needed to confirm this hypothesis. Taken together, while our results in the nucleus accumbens shell region are coincidental, future mechanistic studies could evaluate a role for CaMKII autophosphorylation in enhanced drinking and seeking behaviors that drive individual differences in outbred rats [22,23,24,25,61,62,63].
